# Provider attitudes toward evidence-based practice in autism: a mini-review

**DOI:** 10.3389/frcha.2024.1363532

**Published:** 2024-05-02

**Authors:** Elizabeth A. DeLucia, Samantha M. Harden, Angela Scarpa

**Affiliations:** ^1^ Virginia Tech Autism Clinic & Center for Autism Research, Virginia Tech, Blacksburg, VA, United States; ^2^ Human Nutrition, Foods, and Exercise, Virginia Tech, Blacksburg, VA, United States

**Keywords:** evidence-based practice, autism spectrum disorder, provider attitudes, implementation science, dissemination and implementation, autism

## Abstract

There are many established evidence-based practices (EBPs) for autistic youth which facilitate wellbeing and skill development across a range of domains. However, individuals on the autism spectrum are consistently underserved in mental health settings, limiting their access to these EBPs. Positive provider attitudes toward EBPs may increase their uptake or use. The current mini-review seeks to synthesize the literature regarding attitudes toward EBPs among providers working with autistic youth across a variety of settings (i.e., school, early intervention, and general mental health). Fifteen quantitative studies were included. The majority of studies (*n* = 13, 87%) utilized the Evidence Based Practice Attitudes Scale (EBPAS) or a variation of this scale. Attitudes toward EBPs were primarily used as a correlate or covariate, although some studies reported descriptive statistics of provider attitudes. When available, the reported results suggest that attitudes toward EBPs are moderately positive at baseline, although they vary between provider types. Two studies (13%) examined change in attitudes toward EBPs and suggested that they may be responsive to intervention. However, findings were mixed as to whether attitudes toward EBPs are predictive of EBP use/uptake. Implications and future directions are discussed.

## 1 Introduction

A variety of evidence-based practices (EBPs) have been established to improve outcomes for autistic youth across domains such as mental health, communication, interfering behaviors, adaptive behavior, and social skills (1). Despite the existence of such EBPs, youth on the autism spectrum are continuously underserved in behavioral health and educational settings. Autistic youth face significant barriers to evidence-based healthcare at the provider, organizational, and systemic levels (2). As a result, scholars in the field of autism research have made repeated calls to utilize dissemination and implementation science (DIS) to integrate existing EBPs into settings such as behavioral/mental health clinics, early intervention providers, and schools (3, 4).

While DIS often focuses on organizational level factors to increase uptake of EBPs, some literature has identified individual provider characteristics that are likely to be important to EBP implementation in autism, such as previous training and motivation (5), buy-in and beliefs about own capabilities, and beliefs about program outcomes (6). More work is needed to fully understand the role that provider characteristics may play in adoption, implementation, and sustainment processes. However, both theory and empirical evidence from related fields suggest that provider characteristics are likely to play a role in the improved implementation of EBPs for autistic individuals. The theory of planned behavior [TPB (7)] suggests that individual attitudes, subjective norms, and perceived behavioral control all influence individual intention to act, which in turn influences individual behavior. Applied to DIS, the TPB posits that attitudes toward EBPs should influence provider intention to conduct EBPs, which in turn should influence a provider's behavior of implementing an evidence-based approach.

This theoretical frame appears to be supported by the general literature regarding implementation of an innovation as well as the mental health EBP literature. In the business literature, Frambach and Schillewaert (8) identify that individual attitudes play a role in the adoption of new innovative practices, and Candel and Pennings (9) suggest that the affective component of attitudes is predictive of choosing to implement innovations. In the mental health literature, a study of providers found that attitudes toward specific EBPs were correlated with the degree to which providers used that practice (10). Attitudes toward EBPs are also associated with uptake of components of mental health EBPs such as consultation (11) and routine outcome assessment following a training (12). Attitudes toward research, a closely related concept to attitudes toward EBPs, are also predictive of self-reported EBP use among mental health clinicians (13).

## 2 Research strategy

Despite a robust extant literature on the TPB and attitudes toward EBPs in general, no work has synthesized the literature regarding attitudes toward EBPs in providers working with autistic youth. As such, the current review sought to describe and integrate the literature regarding attitudes toward EBPs among providers, including educators and behavioral or allied health professionals, who work with autistic children and adolescents. The aims of this mini-review are twofold: (1) To characterize and synthesize attitudes toward EBPs among providers who work with autistic youth and (2) to consider key correlates, predictors, and/or outcomes related to EBP attitudes for autism providers.

Studies were included in the current review if they (1) included a measure of provider attitudes toward behavioral health EBPs; (2) included a sample of educators, other school providers, behavioral health providers, or allied health professionals (e.g., occupational therapists), and (3) included a sample of providers working with autistic individuals at least part of the time.

The databases PsycInfo and PubMed were searched in April and May 2023 for terms related to autism, evidence-based practice, attitudes/beliefs, and providers/clinicians/educators. Search terms included: autism, autis*, ASD, autism spectrum disorder, Asperger's, PDD-NOS, attitudes, beliefs, perceptions, opinions, evidence based practice, evidence-based practice, EBP, evidence based treatment, EBT, evidence based, best practice, evidence based intervention, EBI, clinician, teacher, therapist, educator, and provider. Studies meeting the inclusion criteria were retained for synthesis. The Covidence review software was utilized to aid in organization of included studies (14). This search should not be considered exhaustive. Rather, results are intended to provide a broad overview of the scope of the existing literature in order to inform future research on the topic. The search yielded 15 studies, which are included in the current review.

## 3 Results

Refer to Table 1 for details of the following results (15–29).

**Table 1:** Included study characteristics and findings.

Study	Title	Location	*n*	Sample	Measures	Findings
Paynter et al. (15)	Allied health professional's knowledge and use of ASD intervention practices	Australia	156	Psychologists, BA, SLP, or OT	EBPAS	EBPAS requirements scale correlated negatively with amount of time working in autism field
Sam et al. (16)	Employing evidence-based practices for children with autism in elementary schools	Southeastern US state	486	Elementary teachers (SPED, gen ed, admin, SLP)	EBPAS	There was a Group × Time interaction for the appeal domain of the EBPAS, favoring group that received implementation intervention
Paynter et al. (17)	Brief report: Perceived evidence and use of autism intervention strategies in early intervention providers	Australia	71	EI providers including teachers, early childcare providers, and allied health professionals	EBPAS, ATR	The EBPAS was not a significant predictor of intended future EBP use in regressions except for exercise/movement; ATR was a significant predictor of intended future use
Pervin and Hagmayer (18)	Attitudes towards evidence-based practice of professionals working with children and adolescents with autism spectrum disorder in Bangladesh	Bangladesh	150	MH professionals or SPED teachers	EBPAS-36	Providers in Bangladesh reported overall moderately favorable attitudes toward EBP (mean score = 2.70/4.0)
Paynter et al. (19)	Utilisation of evidence-based practices by ASD early intervention service providers	Australia	72	EI providers including teachers, early childcare providers, and allied health professionals	EBPAS	EBPAS requirement and openness scale correlated with knowledge of EBPs and likelihood of using EBP. Requirement subscale correlated with organizational culture variables
Verschuur et al. (20)	Pivotal response treatment: A study into the relationship between therapist characteristics and fidelity of implementation	Netherlands	41	PRT clinicians	EBPAS	EBPAS openness subscale correlated with PRT fidelity, appeal and total score did not
Cheung et al. (21)	The impact of workplace factors on evidence-based speech-language pathology practice for children with autism spectrum disorders	Australia	105	SLPs	Bespoke survey	Most providers viewed EBP as necessary, 41% reported a divide between research and practice, 66% reported that decisions in their workplace were in line with the research
Dyson et al. (22)	Therapists’ adaptations to an intervention to reduce challenging behaviors in children with autism spectrum disorder in publicly funded mental health services	Southern California, US	55	Therapists	EBPAS	Attitudes toward EBP did not predict adaptations made to services
Garrad et al. (23)	From research to reality: Australian evidence-based practice in autism education	Australia	151	Primary school teachers	Evidence Based Practice Innovation Survey	Teachers made decisions to adopt or de-adopt an intervention based on several factors, especially student-centeredness
Stahmer and Aarons (24)	Attitudes toward adoption of evidence-based practices: A comparison of autism early intervention providers and children's mental health providers	US	309	EI and child MH providers	EBPAS	EI providers had more favorable attitudes toward EBPs than MH providers
Paynter and Keen (25)	Knowledge and use of evidence-based practices by community-based early intervention service providers	Australia	99	EI providers including allied health professionals, behavior therapists, teachers, paraprofessionals, and managers	EBPAS	EBPAS openness subscale correlated with EBP use and knowledge, other scales did not
Suhrheinrich et al. (26)	Practice-driven research for statewide scale up: Implementation outcomes of the California Autism Professional Training and Information Network	California, US	1543	Direct service providers and teachers	EBPAS	Providers had moderately high scores on EBPAS openness, requirements, and appeal scales, with lower scores on the divergence scale. People who received EBP training had higher openness scores.
Aranbarri et al. (27)	Examining US Public Early Intervention for Toddlers With Autism: Characterizing Services and Readiness for Evidence-Based Practice Implementation	US	57	Early intervention providers^a^	MPAS	Scores did not differ between providers and coordinators, scores indicated moderate relationship between attitudes toward EBPs and EBP use. Provider EBP attitudes were correlated with organizational factors
Locke et al. (28)	Individual and organizational factors that affect implementation of evidence-based practices for children with autism in public schools: a cross-sectional observational study	Northeastern US	152	K-3 SPED teachers and staff	EBPAS	EBPAS divergence and appeal scales account for 10% of variance in DTT intensity. Openness and requirements scales not associated with any EBP use. No subscales were associated with PRT or visual schedule use
Paynter et al. (29)	Attitudes towards and organizational support for evidence-based practices: A comparison of education and allied health professionals in autism	Australia	236	Allied health professionals and educators	EBPAS	Attitudes were lower for allied health professionals compared to educators on EBPAS total score and appeal, openness, requirements subscales. Attitudes were correlated with organizational factors

ATR, attitudes to Research scale; BA, behavior analyst; DTT, discrete trial training; EBP, evidence-based practice; EBPAS, Evidence Based Practice Attitudes Scale; EI, early intervention; MH, mental health; MPAS, Modified Practice Attitudes Scale; OT, occupational therapy; PRT, pivotal response treatment; SLP, speech language pathologist; SPED, special education; US, United States.

^a^ Study also included early intervention coordinators, administrator, and caregivers but they are not included in presented *N*.

### 3.1 Location and demographics

Seven studies (47%) took place in Australia while six (40%) took place in the United States. One study each took place in Bangladesh (7%) and the Netherlands (7%). Eleven studies reported participant gender; all of these samples were predominately female. Five studies reported participant race; 80% of these included predominately white participants. Mean age ranged from 30.5 (18) to 44.6 (27) among the six studies that reported average age (six additional studies reported age range breakdowns).

### 3.2 Setting/provider types

3,683 providers were participants in the research. Most of these providers participated in studies which included both school and behavioral health providers. 11% of participants were in studies which only included providers in behavioral health and allied health settings, and 21% of providers were in studies that exclusively investigated school provider attitudes.

### 3.3 Measurement strategy

Most studies (87%) utilized the Evidence Based Practice Attitudes Scale [EBPAS (30)] either in its original form (73%) or an adaptation (13%; the EBPAS-36 or the Modified Practice Attitudes Scale, MPAS). The EBPAS is a 15-item measure of provider attitudes toward EBPs. It includes four subscales rated on a scale of 0–4: Requirements (i.e., likelihood of using an EBP if it was required by your organization); Appeal (i.e., likelihood of using an EBP if it is intuitively appealing); Openness (i.e., general open-mindedness to using an EBP) and Divergence (i.e., amount that EBPs diverge or are different from your current practices). The EBPAS-36 (31) is a 36-item form of the measure, and the Modified Practice Attitudes Scale [MPAS (32)] is an 8-item version. Paynter et al. (17) utilized the EBPAS as well as the Attitude To Research Scale [ATR (33)]. The two remaining studies used other questionnaire/survey measures, including the Evidence Based Practice Innovation Survey [EBPIAS (23)] which assesses factors involved in EBP decision making among teachers, and a measure of EBP attitudes, knowledge, and training (21, 34).

### 3.4 Overall attitudes toward EBPs

Most studies did not explicitly report descriptive statistics on EBP attitudes and primarily reported provider attitudes as a covariate or correlate of the likelihood of using EBPs (17, 19, 25), fidelity to EBPs (20) or adaptations to EBPs (22). In studies that did report overall EBP attitudes, attitudes were reported as moderately positive. Average scores ranged from 2.99 to 3.32 out of 4 for all EBPAS subscales except divergence reported in Suhrheinrich et al. (26) Also, EBPAS average score was reported as 2.7 in Pervin and Hagmayer (18).

### 3.5 Group differences in attitudes

Three studies (20%) compared EBP attitudes among provider types. Two of these studies found significant group differences. Autism early intervention providers had more positive EBP attitudes than mental health clinicians working in the United States (24). Additionally, in a study of 261 Australian educators and allied health providers, allied health professionals demonstrated less positive EBP attitudes than did educators (29). In a comparison of early intervention providers and coordinators, there were no group differences on attitudes toward EBPs (27).

### 3.6 Attitudes as a predictor of EBP use

Overall, findings were mixed with respect to the utility of EBP attitudes as a predictor/correlate of outcomes. Several studies identified an association between attitudes and EBP use/fidelity. For example, in a study of early intervention providers in Australia, the Requirement and Openness subscales of the EBPAS were correlated with overall likelihood of using EBPs (19). Another study from the same research team found that in a sample of 99 early intervention providers, the Openness subscale was associated with EBP use and knowledge, while other EBPAS scales did not correlate with EBP use (25). Paynter et al. also investigated the EBPAS's predictive value for the use of several specific EBPs among early intervention clinicians; they found that the EBPAS predicted future use of exercise, an EBP for autistic youth, but did not predict the future use of other EBPs (17). A study of 153 K-3 special education teachers and staff in the northeastern United States found that the Divergence and Appeal subscales of the EBPAS accounted for 10% of the variance in use of discrete trial training (28).

On the other hand, several studies also presented null findings with respect to the association between attitudes and EBP use. Dyson et al. (22) found no association between EBP attitudes and the likelihood of making adaptations to an EBP for autistic youth. Although Locke et al. (28) found a positive association between EBP attitudes and discrete trial training, they noted that there was no association between the EBPAS and use of two other autism EBPs (pivotal response treatment and visual schedules). Furthermore, for many of the studies discussed, particular subscales/components of EBP attitudes were most associated with outcomes, rather than an association between outcomes and a provider's overall EBP attitudes.

### 3.7 Altering attitudes toward EBPs

Two studies investigated whether intervention could change individual provider attitudes toward EBPs. These interventions included approaches such as training, goal setting, and coaching/feedback, and attitudes toward EBPs improved when these DIS interventions were implemented. For example, Sam et al. (16) conducted a study in which schools were assigned to a professional development model called the National Professional Development Center (NPDC) or to services as usual. Schools in the NPDC condition used a four-part process which included focus on overall classroom quality, designation of measurable student goals, matching student goals with appropriate EBPs, and coaching/feedback. The authors found a group by time interaction effect on the appeal domain of the EBPAS, such that attitudes toward intuitively appealing EBPs improved from pre-test to post-test, only for the NPDC condition. Another study investigated EBP attitudes in providers who had vs. had not participated in the California Autism Professional Training and Implementation Network [CAPTAIN (26)]. CAPTAIN is a network designed to develop dissemination plans and provide training on EBPs throughout California. Results indicated that individuals who had attended CAPTAIN training displayed higher levels of Openness on the EBPAS, indicating more willingness to implement EBPs into their practice, relative to the control group.

## 4 Discussion

The included studies suggest a mixed literature regarding autism provider attitudes toward EBPs. However, they provide some evidence for the importance of attitudes as an implementation factor, consistent with both the TPB and existing DIS frameworks. Overall, the reviewed literature suggests that autism providers have moderately positive attitudes toward EBPs, although there is room for improving attitudes. Interestingly, the vast majority (87%) of included studies used a version of the EBPAS to measure attitudes. Future psychometric research would do well to evaluate the use of this measure specifically with autism providers, to better understand how the measure applies for this population. Additionally, there is some evidence that attitudes toward EBPs are malleable in the two studies that examined interventions, with improved attitudes after DIS strategies including feedback, training, and goal setting (16, 26). This finding is encouraging, as it suggests that there is potential for modifying EBP attitudes through intervention.

Another important question to consider, then, is whether attitudes are a meaningful construct in terms of predicting EBP use/fidelity in providers working with autistic youth. DIS researchers identify intervention acceptability and appropriateness, concepts closely related to attitudes, as key implementation outcomes (35–37), suggesting their importance in implementation success. Within the autism literature specifically, qualitative research has suggested that providers perceive EBP attitudes (at the provider and leadership level) as important in influencing implementation (38). Several studies in the current review supported this idea, finding that EBP attitudes are correlated with EBP use (17, 19, 25, 28), and one study identified an association between EBP attitudes and implementation fidelity (20). Yet, other studies found no association between attitudes and EBP use (17, 22, 28). There was considerable variability in the settings and types of autism providers involved in the studies, which could explain the range of findings. Overall, the present review highlights that while EBP attitudes have promise as a predictor of implementation outcomes, there is an overall lack of consensus in the literature regarding the impact of provider EBP attitudes. While the wider literature has established that leadership and organizational factors are key to implementing EBPs in autism practice (38–40), this review underscores the need for further research to clarify the mixed landscape regarding individual provider attitudes toward EBPs.

It is also important to consider the variety of practices which fall under the EBP umbrella in the autism literature. DIS has firmly established the importance of contextual factors when implementing interventions (41–44), and a wide range of contexts are at play in autism intervention due to the interdisciplinary nature of autism services. In recent years, the COVID-19 pandemic has resulted in a shift to telehealth services for many health professionals, including those who work with autistic individuals (45–47). The impact of this contextual factor on providers' attitudes toward EBPs is not well understood, and future research should investigate this further. Gabellone et al. (46) developed a questionnaire examining provider attitudes toward using telehealth with autistic patients; this measure may provide a useful starting point to consider provider attitudes toward conducting EBPs over telehealth.

Additionally, attitudes toward EBPs as a broad construct may be significantly different than attitudes toward an individual EBP. The clearest example of this possibility is applied behavioral analysis (ABA), an umbrella term which can encompass a range of autism EBPs. ABA has been subject to increasing scrutiny over the last decade, with many autistic individuals arguing that the outcomes ABA seeks to create are akin to “normalization” of autistic people and do not target factors that are most important to autistic individuals themselves (48). This perspective highlights an important consideration within the context of the current review: there is substantial heterogeneity in the outcomes targeted in autism EBPs. Such variability highlights the importance of using participatory designs that consider autistic perspectives when conducting future research on attitudes toward and use of EBPs.

### 4.1 Implications for implementation science

The present review has implications for implementation science in general and within the autism field. Given the emerging evidence provided here for malleability of attitudes, it is recommended that implementation scientists consider implementation strategies, such as those identified by Powell et al. (49) (e.g., educational outreach, local consensus discussions, providing ongoing consultation) with the explicit goal of altering attitudes toward EBPs in general and toward specific EBPs. This work can subsequently measure downstream effects on adoption decisions, implementation, and sustainment. If effective, using DIS strategies to alter attitudes may serve to decrease translational lag time and ultimately improve access to services.

The process of changing attitudes should be done in collaboration with the community, preferably through participatory research (50, 51). In addition to including autistic individuals in research partnerships, it may also be beneficial to include caregivers for autistic people, given that many EBPs include a family-mediated approach (1). Models such as the integrated research-practice partnership process model (52) may aid in this approach. In addition, applying a health equity lens to existing frameworks, as described by Fort et al. (53) may prove important particularly for historically underserved populations. When considering attitudes, it is critically important for implementation scientists to consider the role and perception of researchers by the community. Partnering with community groups is therefore a necessary step. As such, the implementation strategy of identifying champions (46) may prove useful, particularly if attitudes are to be targeted.

It is further recommended that implementation scientists also consider provider attitudes toward a *specific* EBP to determine whether modifying attitudes toward a particular approach changes uptake. General mental health literature suggests that EBP attitudes can vary by EBP type (10). In the short term, implementation scientists should consider measuring both attitudes toward EBPs broadly as well as attitudes toward a specific EBP, in order to clarify the role that attitudes toward EBPs broadly and attitudes toward specific practices play in the implementation process [e.g., Kemp et al. (54)].

## 5 Conclusion

The current review offers a synthesis of existing literature regarding attitudes to EBPs among autism providers and suggests several future research directions. Overall, EBPs are generally viewed favorably, though attitudes may differ by provider type, and the research is mixed on whether EBP attitudes impact uptake by autism providers. Future implementation science, ideally with participatory research designs, should continue to consider autism provider EBP attitudes and the factors that influence their impact on EBP use with autistic youth.
